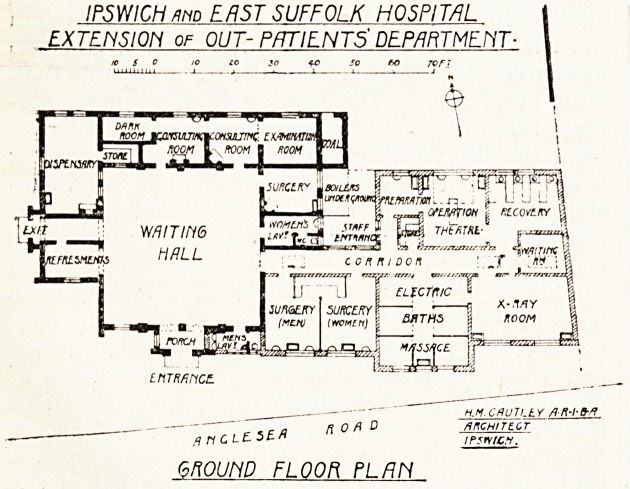# The Ipswich and East Suffolk Hospital

**Published:** 1912-12-07

**Authors:** 


### The Ipswich and East Suffolk Hospital.

The plan we publish shows an extension of the out-patient block which was built in 1904, being a gift of a former honorary member of the medical staff, Dr. J. H. Bartlet.

The new building, shown black on plan, comprises an entrance porch, with a large waiting hall, two consulting rooms, examination room, dark room, dispensary, refreshment room, surgery, lavatories for male and female patients. The operation work now done in the room marked "Surgery" will in future be carried on in the theatre in the north side, which has adjoining it a preparation room and recovery room, the preparation room being used for the administration of anæsthetics. The surgery is so arranged that, while the compartments for each sex are separated by a marble partition, the stands for dressings, etc., are common to the two rooms. The $x$-ray room is arranged primarily for a treatment room, but can be used for radiography if necessary. It is designed to contain two sets of apparatus, but provision is made for only one at present. Adjoining the $x$-ray room is a room for electric baths and massage, containing four compartments, each with a curtained front.

IPSWICH AND EAST SUFFOLK HOSPITAL

EXTENSION OF OUT-PATIENTS' DEPARTMENT.

GROUND FLOOR PLAN

The architect for these alterations is Mr. Munro Cautley, A.R.I.B.A., of Ipswich.

## Figures and Tables

**Figure f1:**